# Morbid Impulses

**Published:** 1874-05

**Authors:** H. R. Bigelow

**Affiliations:** Hartford Conn.


### Morbid Impulses.

By H. R. Bigelow, M. D., Hartford Conn.

Every one in his life experience can recall to himself occasions in which an idea could not be dismissed from his mind by any volitional effort, being realized in action in defiance of his will and better judgment.

An essayist, evidently an Englishn an,* writing not long since upon this subject, related an instance which occured in his own life. It was at a party given by Mrs. Cantelope, the pretty daughter of an insignificant pork merchant. She marrying the younger son of a rich man became the representative of a powerful English family. Her "pride of place" and forcible manner of canvassing the claims of parrence families were such that our English friend was seized with a tyrannical impulse to say an insolent thing, and it was only by hastily retiring from the assembly and rushing to his own quarters, that he rescued himself from shouting out, "Madam, your Father was a little Pork Butcher—you know he was." Full well he knew the ignominy which would follow such an explosion—the rudeness of the remark was apparent, yet was the dominant power of the idea such, that his *Will* became powerless, and to escape a greater evil he must needs run away.

Once upon an occasion of extreme solemnity, none other than the funeral of a near and dear relative, some ludicrous stimulus excited in me an uncontrollable desire to laugh. In vain I tried to realize the sadness of the time—to remember the holy traits of the departed one—to sympathize with the grief of the immediate relatives. I sat on a rack of unrest, the perspiration hung in drops upon my forehead. I must laugh or go mad, and so, just as the corpse was being raised by the mourners, laugh I did, yet did the impulse seem in no way lessened in power by the horror stricken faces around me.

A friend of mine, in whom the neurotic temperment is strongly marked, has constantly the morbid desire to do queer and reprehensible things. He is in daily fear of himself. His watch is an unfailing source of annoyance, as the sight of it is always accompanied by the desire to introduce it forcibly to the head of his nearest neighbor. A bridge is a thing of horror, suggesting to

*Harper's Monthly Magazine.

him suicidal ideas. At one time, suffering as he was from an attack of hysteria, he would bark like a dog, crow, and mew, in imitation of similar sounds which had reached his ears through the open window.

Even now, at the age of thirty, he has so little confidence in his ability to fight against the impulse successfully, that he never leaves the house alone, lest he renders himself amenable to the law by giving expression to the inward self-asserting idea. How often, for instance, have we seen the suddenly excited idea of the ludicrous give rise to involuntary and unexpected laughter. In this way I am sometimes forced to smile when the passages of the play are of the most pathetic nature.

Who, standing upon some dizzy height, has not felt an unpleasantly controlling desire to cast himself down—or who, gazing down upon the ever shifting scenes of a placid river, has not longed to fathom the mysteries of its depths? A single plunge—a musical ripple—nothing more. Who has not at sometime aroused himself to the fact that his hand was unconsciously keeping time to the strains of an orchestra, or that he was humming the accompaniment of a difficult vocal passage?

All of us have experienced these sensations, but few can account for them. Can they be explained on physiological or psychological grounds? Are they spiritual or material manifestations? How closely allied to insanity may they be? How far will they act as extenuating circumstances in the commission of a crime?

Let us enter into their pathology together. And to this end, we must first understand a little of the nervous centres in which these impulses may arise; the centres which give us mental action, sensation, sensory-motor and reflex action. These are;

1. *Primary* or *Ideational* centres, constituted by the gray matter of the convolutions of the hemispheres.

2. These are the *Secondary* nervous centres, or *Sensational* centres, constituted by the collection of gray matter that lies between the decussation of the pyramids and the floors of the laterial ventricles.

3. There are the *Tertiary* nervous centres, or centres of *Reflex Action* constituted mainly by the gray matter of the Spinal Cord.

4. There are the *Quarternary* nervous centres, as we might call them, belonging to the Sympathetic System.*

Now although each centre is subordinate to the centre immediately above it, each is capable of determining certain actions without the intervention of its supreme centre, an independent local action being entirely compatible with the control of a supreme authority. The spinal centres are subordinate to the sensory centres, these latter to the controlling action of the cerebral hemispheres—more especially to the action of the will. The Spinal Cord consists of those nervous centres which give rise to reflex

*Maudsley. Physiology and Pathology of Mind.

action, and by its operation causes the unconscious movements which form a large part of human activity, and which are due to the independent power of re-action of these centres. Infantile movements are reflex to impressions without. The accumulated experience of generations has stamped within its centres a veritable Memory, so that in response to certain stimuli, the same results will always be effected. The force responsive to a stimulus, may be entirely expended outward through an efferent nerve, producing muscular action, or only partially so, the residua being conveyed upward to the sensory centres causing sensations. The *Secondary* nervous centres, *Sensory Ganglia,* included among other things, the ganglionic nuclei of different senses. In many of the lower animals the brain consists of nothing more than these sensory ganglia, with centres of motional reaction. In answer to an appropriate stimulus, the whole force of the centre may be expended outwards on a motor nerve, and movements in harmony with the sensation result, in the same way that involuntary movements take place in the Spinal Cord *without* sensation ; or a part of the force may be extended upward to the primary or ideational centres. The cells also are centres of independent re-action, and may give rise to a series of reflex actions of their own. Most of these actions are automatic, the acquisition of generations. The instinctive actions of animals are consensual. The sucking of the infant, some of the movements of mastication, the adjustment of the eye to certain distances, fall under the head of consensual acts. Sensations are not innate but acquired. "The complete sensation is built up in the nervous centre, from the residua left behind by like sensations, and the sensation of a cultivated sense thus sums up, as it were, a thousand experiences, as one word often contains the accumulated acquisition of generations." Thus briefly, but sufficiently, we have glanced over the subordinate centres, until we have arrived at the *Supreme, Primary* or *Ideational* centres. Upon these centres, the ablest psychological and physiological investigations have been expended, and will continue to be thus expended, until, in the purification of future ages, men's minds shall have attained that trained habit of inquiry, so absolutely necessary in mental pathology. As I have shown elsewhere,* the mere metaphysical, subjective introspect will avail us no purpose, for the abstraction which is necessary for the performance of conscious scrutiny withdraws from mental freedom that which is necessary for its perfected entity and creates abnormal existence in the object to be analyzed by self-consciousness. It is now universally admitted that the nerves cells of the gray corticle layers of the hemispheres of the brain, are the nervous centres of idea. The molecular changes occurring in these millions of cells give rise to a force which we call Mind. Mind, then, is perfected force generated by the supreme or ideational centres, represented in the

*Hamlet's Insanity.—Chicago Medical Journal Sept., 1873.

convolutions and corticle cells of the brain, not a spiritual vagary, an abstract, incomprehensible agent, but a natural force, situated in the Brain. Now, as in the ganglionic cells of the sensory centres and of the Spinal Cord, so also in these corticle cells of the hemispheres is there independent re-action. An active idea may pass outward even in direct defiance of the will and manifest itself in some bodily movement. An idea may arise and produce movement without any consciousness of it. Here, then, we have the explanation of the Morbid Impulse. When the impulse becomes dominant, asserting itself despite the will, then it is that the person is pronounced insane. The mere existence of the *fixed idea*, so long as it be controlled by volition, is in no wise an abnormality. When the hemispherical cells cease to re-act upon each other harmoniously, when an idea prolongs its tension so as to "tyrannize over the understanding and become an absorbing entity," illusions and delusions result. A man in this condition of mental erythism, acting under a delusion would not be amendable to law only in so far as his confidence in a proper asylum would be demanded. The modus operandi by which an idea becomes excited and active in this: The necessary external stimulus applied to the sensory ganglia is expressed outwardly as pleasure or disgust, while that residua furnish to the well balanced mind the stimulus which was necessary to excite the particular idea in one of the numerous corticle cells. Just what stimulus was needed, and just what idea would obtain from its application, are the lessons stamped on the mental growth by the experience of generations. The nervous action may become weakened by the vicious transmission of heredity, or the integrity of the nervous vitality of the centres may be upset by injurious practices. Hence, in such abnormal minds, the stimulus excites the sensory ganglia in extreme degree. The sight of a river, the impression being conveyed by the eye to the optic centres, to be realized as sensation, creates an exaggerated feeling of cold, of fifth, etc. These ganglia, themselves lowered in vitality, send upward to the brain false impressions. The unwonted, abnormal stimulus calls out a diseased idea, which being freed from the scrutiny of a healthy Will, becomes predominant and is often realized outwardly in most painful ways.

How does *Will* control and regulate the mental action? Maudsley's definition will furnish the answer. "*Will* is the desire or aversion sufficiently strong to generate action upon reflection." But reflection is the just balancing of ideas; hence this process cannot go on if one idea is allowed to become supreme.—*Cincinnati Med. News.*

